# Sheffield Adaptive Patterned Electrical Stimulation (SHAPES) Therapy for Post Stroke Arm spasticity: study protocol for a 3-arm, a partially blinded, randomised controlled trial

**DOI:** 10.1186/s12883-024-03635-x

**Published:** 2024-11-09

**Authors:** Ali Ali, Avril D. McCarthy, Mark Reeves, Jamie Healey, Louise Moody, Adewale Adebajo, Tim Good, Simon Dixon, Kathleen Baster, Wendy Tindale, Krishnan Padmakumari Sivaraman Nair

**Affiliations:** 1 Sheffield Institute of Translational Neurosciences, University of Sheffield, Sheffield, UK; 2 Combined Community and Acute Care Group, Sheffield Teaching Hospitals NHS Foundation Trust, Sheffield, UK; 3 Clinical Engineering, Sheffield Teaching Hospitals NHS Foundation Trust, Sheffield, UK; 4 NIHR Devices for Dignity MedTech Cooperative, Sheffield Teaching Hospitals NHS Foundation Trust, Sheffield, UK; 5 School of Health and Related Research (ScHARR), Faculty of Medicine, Dentistry and Health, The University of Sheffield, Sheffield, UK; 6 University of Coventry, Coventry, UK; 7 Department of Rheumatology, Barnsley Hospital NHS Foundation Trust, Barnsley, Barnsley, UK; 8 Department of Neurosciences, Sheffield Teaching Hospitals NHS Foundation Trust, Sheffield, UK

**Keywords:** Stroke, Spasticity, Electrical stimulation, TENS, SHAPES, RCT

## Abstract

**Introduction:**

Post stroke elbow spasticity (PSES) affects over a third of individuals following stroke and negatively impacts on functional recovery, comfort and quality of life. Drug therapies have limited efficacy and unwanted side effects, botulinum toxin, although effective, is costly, and conventional electrical stimulation therapies are limited long term by habituation. We aim to investigate the efficacy of Sheffield Adaptive Patterned Electrical Stimulation (SHAPES), that delivers temporally and spatially varying pattern of electrical stimulation, against transcutaneous electrical stimulation (TENS) and standard care at reducing PSES.

**Methods and design:**

Overall, 297 people with PSES will be randomised (1:1:1) to one of 3 arms: Standard care (no electrical stimulation), TENS (conventional patterned electrical stimulation) or SHAPES (adaptive patterned electrical stimulation). Both SHAPES and TENS are delivered using a specially designed electrical stimulation sleeve used for 60 min each day for 6-weeks. Outcome measures are completed at baseline, end of treatment (EOT 6 weeks) and then 6-weeks, 12-weeks and 24-weeks after the end of treatment. Efficacy will be determined based on the proportion of participants experiencing meaningful improvement (18%) in the 7-day Numerical Rating Scale (NRS-S) for PSES, compared between both intervention arms and standard care, and between the two intervention groups. Measures of arm motor function (Action Research Arm Test, MRC scale), and quality of life (SQoL-6D, EQ-5D) will also be measured along with a parallel health economic evaluation.

**Discussion:**

The results of the SHAPES trial will inform management of elbow spasticity after stroke. The SHAPES intervention is a low cost, self-administered intervention for the management of spasticity that can be used repeatedly, and if found to be more effective than TENS or control has the potential to be widely implemented in the UK NHS healthcare setting. Furthermore, despite the wide use of TENS in the management of spasticity, this study will provide critically required evidence regarding its efficacy. The trial has been registered with the ISRCTN registry (ISRCTN26060261).

**Supplementary Information:**

The online version contains supplementary material available at 10.1186/s12883-024-03635-x.

## Introduction

There are over 1 million stroke survivors in the UK, with over 100,000 new cases each year [1] making it one of the leading causes of adult death and disability. An ageing and more multi-morbid population mean the number of stroke survivors aged 45 years or over is expected to rise by 123% over the next 20 years [2]. Long term upper limb impairment affects 40–50% of people in the chronic phase of stroke [3] and can manifest as weakness, apraxia, sensory disturbance, or spasticity.

Spasticity is a velocity dependent increased resistance to passive stretch and hyperactivity of stretch reflexes [4] that can result in pain, spasms, reduced range of movements, and ultimately contractures. Arm spasticity affects over a third of people with arm weakness a year after stroke [5] and impacts greatly on return to function and quality of life [6]. Treatment strategies for spasticity exist, however they each have limitations that mean spasticity is still a difficult complication to manage. Management of triggers known to aggravate spasticity and traditional stretching therapies remain the cornerstone of spasticity management but can be labour intensive if delivered by trained therapists or therapy assistants. Oral anti-spasticity medications, such as Baclofen in high doses can be effective, however, they have considerable side-effects, including sedation, weakness, confusion, and depression. Medication adherence with oral antispasticity drugs is poor, typically between 15% and 50% [7]. Botulinum toxin injections can relax spastic muscle for up to 12 weeks, without side-effects of global weakness or sedation, however they can take several weeks to become effective, and are resource heavy in terms of the need for repeated injections delivered in specialist clinics. This can have the effect of at least doubling direct care costs of managing spasticity [8]. Improved therapies for spasticity are much needed.

One such alternative therapy includes neuromuscular electrical stimulation (NMES) techniques, such as transcutaneous electrical stimulation (TENS). Stimulation of peripheral sensory nerves is thought to induce neuroplastic change in spinal cord pathways, leading to inhibition of spinal motor neurones [9]. TENS is an attractive option, given its low cost and ease of administration. Meta-analyses of randomised controlled trials (RCTs) evaluating the effects of TENS for spasticity demonstrate moderate improvements in spasticity severity and limb range of motion compared to control [10] but these improvements are often short lived. The recent NICE guidelines on stroke rehabilitation in adults 2023 recommends research on cost effectiveness of NMES therapies like TENS [11].

TENS often use single channel, single strength, fixed duration modes of stimulation to which the nervous system may get habituated. Habituation is the reduction in neuromuscular response to a repeated stimulus and is associated with a decrease in transmitter release from synaptic afferent nerve terminals, due to inactivation of calcium channels in the presynaptic membrane [12]. One way to try and mitigate the effects of habituation is to alter the pattern of electrical stimulation delivered. Indeed, alternating the frequency of electrical stimulation delivered by TENS had the effect of reducing habituation and improving duration of effect when delivered for pain management compared to non-modulated TENS [13]. With this in mind, the department of Clinical Engineering at Sheffield Teaching Hospitals, in collaboration with neurorehabilitation clinicians, underwent a series of co-development stages to co-design the Sheffield Adaptive Patterned Electrical Stimulation (SHAPES) therapy with people with lived experience of stroke [14]. In an initial proof of concept study, 10 patients with elbow flexor spasticity, Modified Ashworth Score (MAS) ≥ 2, underwent both TENS or SHAPES stimulation for 60 min just below the threshold for motor activation, in a randomised order, a week apart. Immediately following stimulation, spasticity (MAS and visual analogue scale 0-100) improved following both types of stimulation but persisted for up to an hour following SHAPES stimulation only [15]. Subsequently a single blind, crossover, randomised controlled feasibility trial recruited and randomised 16 patients with post stroke elbow spasticity (PSES) to receive either TENS or SHAPES, delivered once daily for 60 min, for 4 weeks before switching to the other treatment modality after a 2-week washout period. The intervention, delivered by patients themselves or care givers was safe, acceptable, and feasible [16].

The Sheffield Adaptive Patterned Electrical Stimulation (SHAPES) study aims to assess the efficacy of SHAPES and TENS electrical stimulation therapy for PSES. It will also provide a health economic analysis to guide implementation in the UK healthcare setting. The protocol is presented according to the Standard Protocol Items: Recommendation for International Trials checklist [17].

### Study objectives

#### Primary objectives

1. To determine whether SHAPES and/or TENS interventions lead to a greater proportion of participants with PSES experiencing meaningful improvements in spasticity (NRS-S change > 18% compared to usual care) after 6 weeks of treatment.
2. To determine whether the SHAPES intervention leads to a greater proportion of participants with PSES experiencing meaningful improvements in spasticity compared to TENS after 6 weeks of treatment.

#### Secondary objectives

1. To determine whether any differences in efficacy relating to spasticity reduction described above between groups persists at 6 weeks, 12 and 24 weeks after end of treatment.
2. To investigate between-group differences in changes to other measures including Modified Ashworth Scale (MAS), arm motor function (Action Research Arm Test, ARAT) and strength (Medical Research Council, MRC strength scale), spasticity impact (Leeds Arm Spasticity Impact Scale, LASIS) and European Quality of Life Scale 5D-5 L (EQ-5D) and spasticity specific quality of life scale (SQoL-6D).
3. To develop a health economic model to inform a cost-effectiveness analysis for the UK healthcare system.

## Methods and design

### Design

A partially blind, multi-site, 3-arm randomised control trial, evaluating the change in elbow spasticity after six weeks of intervention measured by NRS-S and with the key outcome of interest being the proportion in each group that achieve an improvement of 18% in 7-day average NRS-S between the two time points. The investigation compares the efficacy of the SHAPES stimulation to TENS stimulation and both compared to usual care at different time points.

### Study setting and timeline

The trial is being conducted in South Yorkshire, UK, that includes hospitals delivering care for approximately 1 million people in Sheffield, Rotherham, and Doncaster. Study assessments and procedures are taking place at the NHS facilities of Sheffield Teaching Hospitals NHS Foundation Trust, UK. The study opened for recruitment in June 2023 and will recruit to December 2024.

### Participants

We calculated the sample size using data from our previous study [15]. The sample size calculation for a two group t-test for equal means showed that to conduct a three arm randomized control trial of SHAPES, TENS and ‘usual care’ control, we would need 66 subjects per group (total 198 participants) to have 90% power to demonstrate a MCID reduction of 18% in NRS at the 1% significance level to allow for multiple testing. In our feasibility study 67% of participants showed good adherence with the intervention and 73% with recording of NRS. We will recruit a total of 297 patients with PSES to the study who are being randomised to one of the 3 arms. We aim to achieve a minimum of sixty-six (66) participants to adhere to and complete the protocol in each group with good adherence to the intervention schedule and a minimum of 4 of 7 daily NRS-S recording.

Patients will be eligible to participate according to the inclusion and exclusion criteria listed in Table 1. Patients must be between 2 and 26 weeks from the onset of stroke and have any degree of arm spasticity (MAS ≥ 1) with at least mild weakness of the elbow flexors (MRC ≤ 4). Those with implantable electrical stimulation devices, participating in other interventional upper limb studies, or with pre-existing musculoskeletal of neurological disorders affecting elbow movement or spasticity are excluded. Aphasia and cognitive impairment are not exclusion criteria as long as patients can understand the intervention and complete study outcome measures.

Potentially eligible participants are identified by treating clinicians working in the stroke and rehabilitation services at Sheffield, Rotherham and Doncaster. Treating clinicians provide potential participants with the study information and obtain their consent to contact. Central researchers at Sheffield will call those consenting to be contacted via telephone to arrange a screening visit if they are willing to participate.

### Study procedures

At the screening visit, participant written informed consent is obtained by study researchers, eligibility confirmed by a medical doctor of the study team, demographic data recorded, and a baseline assessment of healthcare utilisation recorded. The participant is trained on recording daily NRS-S for 7 days (baseline assessment), recorded either using a dedicated smartphone ‘App’ or as a paper diary to suit their preference. The average of which (minimum 4 of 7 days scores) forms the baseline 7-day NRS-S score, completed prior to attending Visit 2. The participant is then be randomised (1:1:1) to either:

- SHAPES
- TENS
- Usual care

**Table 1:** Study inclusion and exclusion criteria

**Inclusion Criteria**
1. Age 18 to 100 years
2. 2-26 weeks after stroke
3. Weakness of elbow extension of MRC grade 4 or below
4. Spasticity of elbow, of grade-1 or more on the Modified Ashworth Scale (MAS) of elbow flexion
**Exclusion Criteria**
1. Dermatological, rheumatologic or orthopaedic illnesses of the affected arm interfering with elbow movement
2. Pre-existing severe systemic disorders like cardiovascular disease, active cancer or renal disease, end stage pulmonary or cardiovascular disease, psychiatric illness including severe alcohol or drug abuse and depression
3. Expected inability to perform the baseline assessments such as in those with severe aphasia or dysphasia
4. Severe tactile hypersensitivity
5. Participation in other, spasticity related studies
6. Within 12 weeks of receiving elbow flexor Botulinum toxin injections
7. Uncontrolled epilepsy
8. Any form of implanted electrical / electronic device
9. Pregnancy
10. Inability to provide informed consent
11. Pre-existing upper limb spasticity
12. Previous acute contact dermatitis and/or known allergy to acrylates.
13. Within 2 weeks of receiving, or planned future use of, other forms of electrical stimulation to the elbow flexors or extensors

Randomisation is facilitated by blinded researchers using Castor EDC clinical trials management software, which employs validated variable block randomisation model. Its algorithm is constructed in such a way that randomised inclusions are divided across groups in variable block sizes, which is intended to ensure true randomness during the allocation.

At the second study visit, other baseline outcome measures are completed. Participants in the SHAPES or TENS arms receive a specially designed upper limb electrical stimulation system (ShefStim APS) and are trained on how to apply and use the device each day during the intervention period. All devices are configured to the allocated TENS or SHAPES arms of the study using a QR code to maintain blinding. Participants then deliver the intervention at home independently or with the help of a care giver for 6 weeks. After 2–3 weeks all participants are contacted to check that they are managing the NRS-S diary, with those in the device arms also receive a safety / device review with the study team. At the end of the 6-week intervention period they return for end of treatment outcome measures that also include an assessment of healthcare utilisation. These assessments are repeated at 6 weeks, 12 weeks and 24 weeks after the end of intervention period. All participants are invited to undergo experiential interviews, providing qualitative data on intervention use. While participants and clinicians facilitating the treatment checks will not be blinded to the control group, outcome measures will be undertaken by researchers blinded to treatment allocation, while all participants and researchers undertaking outcome measure assessments will be blinded to type of electrical stimulation.

### Interventions and control

**Fig. 1:**
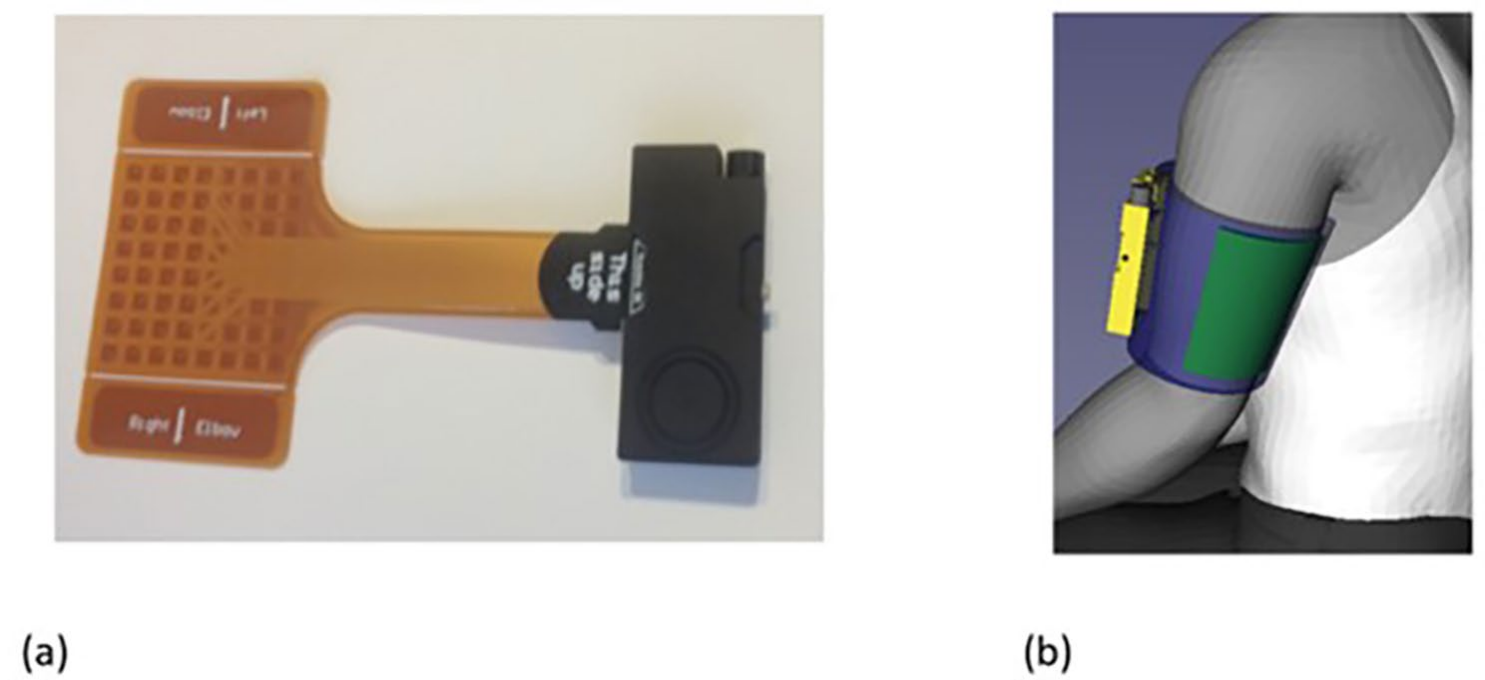
ShefStim APS Stimulator with connected electrode array (**a**). SHAPES system applied to the upper arm (Shefstim APS stimulator box in yellow, electrode array in green, arm sleeve in blue) (**b**)

Participants in the device intervention arms are provided with an electrical stimulator system, (Fig. 1) to use every day for 60 min, for the 6-week intervention period. The system consists of a small box containing the stimulator electronics (ShefStim APS), linked to a flexible array of 64 electrodes (arranged as 8 rows by 8 columns), overlaid with a bespoke biocompatible patterned hydrogel layer (SEKISUI ST-GEL, NR-SO320/100, SEKISUI KASEI CO.LTD.), worn over the upper arm with a bespoke flexible sleeve. It sends electrical impulses via the array of 64 individually controllable electrodes.

Although programming of each electrode is fully flexible, for this study the two configurations are:

#### TENS

The central 16 array electrodes (4 × 4) are activated simultaneously with a 1s rising edge ramp building up to 4s of stimulation at a pulse repetition frequency of 100 Hz and with a pulse width of 250µs, followed by a 1s falling edge ramp and then a 4s off period. The stimulation pattern is then repeated.

#### SHAPES

Each horizontal row of 8 electrodes on the array is sequentially activated for 0.3s at a pulse repetition frequency of 50 Hz and with a pulse width of 250µs. After all 8 rows of electrodes have operated the stimulation pauses for 2.5s before the sequence is repeated.

The participants in the usual care arm do not receive any device. The systems are programmed following randomisation allocation, using a QR code that is scanned onto a stimulator remote control (repurposed Alcatel 1c smartphone) and delivered via Bluetooth LE to the stimulator box. The participant’s mid-bicep circumference is be measured and the oversized sleeve cut to size to ensure that a personalised sleeve fit is achieved. When applied, the biocompatible hydrogel layer should contact the skin overlying the extensor aspect of the affected upper limb, over the triceps area. During the setup of the ShefStim APS (for those in the SHAPES or TENS arms of the study) the stimulation intensity (current) is gradually increased until the underlying muscle begins to twitch (motor threshold, MT). If the participant experiences any discomfort from the stimulation, the intensity is limited to the highest that is comfortable for them. The stimulation level is then set to 90% of the MT or, if the MT was not reached, 90% of the maximum comfortable level. This intensity is recorded at baseline and becomes the default level for home use. Participants are trained on how to apply the sleeve and, if necessary, adjust the stimulation intensities (within clinically set levels) for comfort.

During the intervention participants can receive all usual cares for spasticity management provided by the NHS (e.g. botulinum toxin, oral agents, stretching exercises etc.). This will be recorded and compared between the 3 groups and adjusted for in the statistical analysis.

### Outcome measures

Clinical examinations of outcome measures are highlighted in Table 2, are collected at baseline, end of treatment (6 weeks), and then 6, 12 and 24 weeks after the end of treatment. These are performed by clinicians with appropriate experience in their application. The primary outcome measure is the.

**Table 2:** Study Outcome Measures

**Primary outcome measure** (End of treatment – 6 weeks)	Mean NRS for 7 days.
**Secondary outcome measures**	MAS
	MRC
	Leeds Arm Spasticity Impact Scale
	ARAT
	EQoL 5D-5 L
	SQoL − 6

averaged 7-day NRS-S. This is a simple patient reported level of elbow spasticity where 0 = no spasticity and 10 = worst possible spasticity. This is recorded daily either on paper report forms or digitally according to patient preference. Its validity and reliability in stroke is established as is the minimally important clinical difference (MCID) [18]. Through co-design we have adapted the NRS-S to enable easier completion amongst those with cognitive impairment and aphasia (Fig. 2). The MCID for the NRS-S is a change of ≥ 18% (i.e. 2 points) from baseline to follow up assessment. ^17^ The primary endpoint for this study is the proportion of participants experiencing this MCID improvement in each group. Averaged 7-day NRS-S is calculated at baseline, end of intervention (6 weeks), and then at 6 weeks, 12 weeks and 24 weeks after the end of intervention (Fig. 3).

**Fig. 2:**
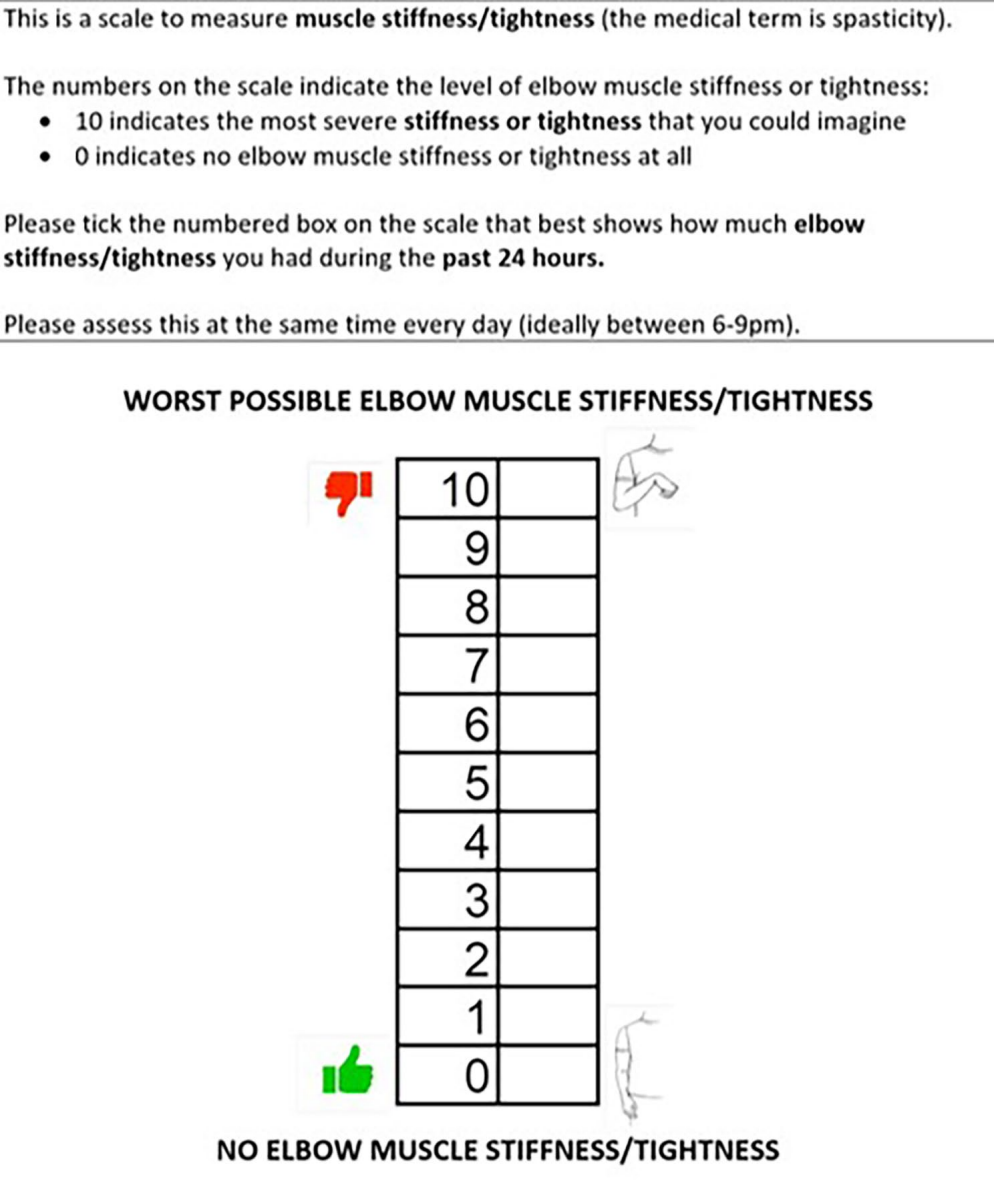
Adapted Numerical Rating Scale for Spasticity (NRS-S)

**Fig. 3:**
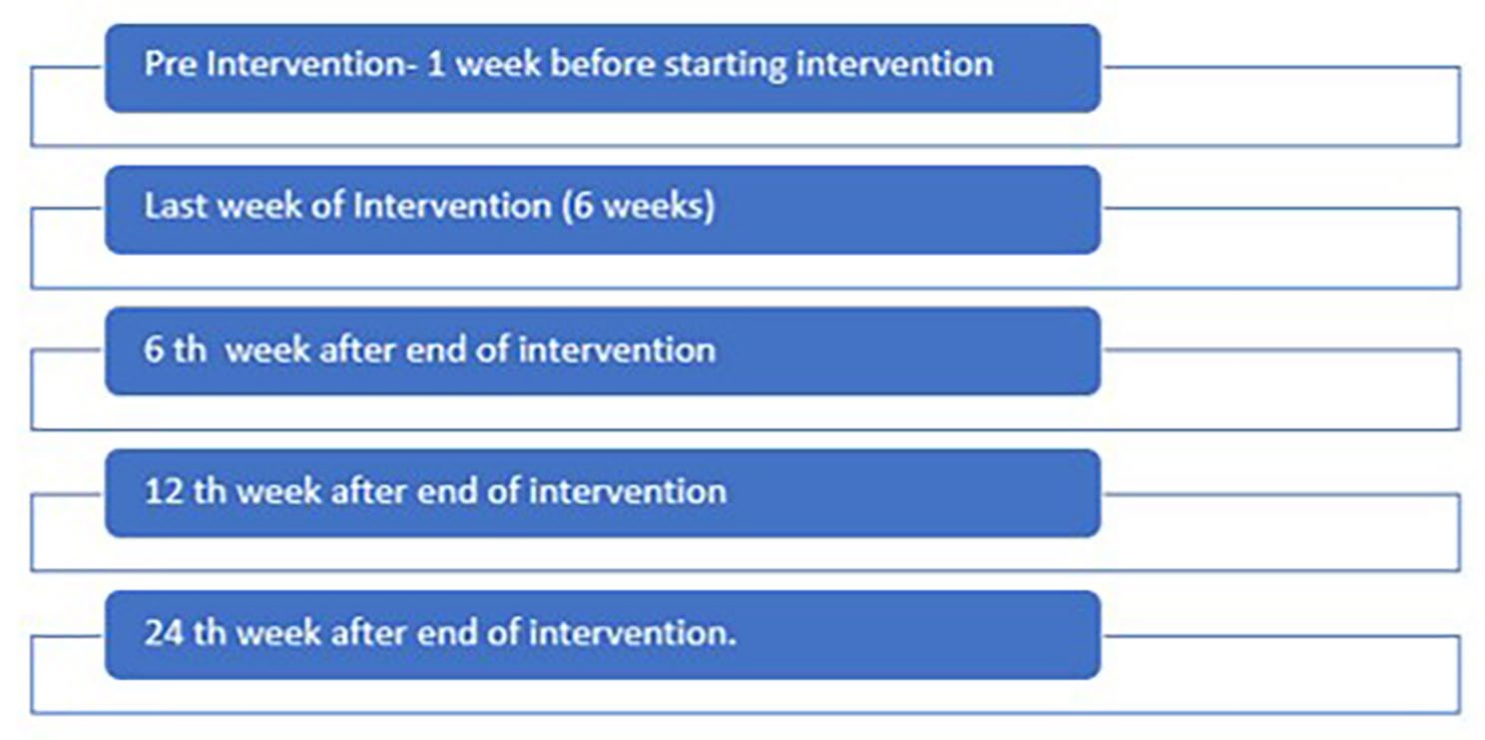
Schedule for 7-day average NRS-S recording

Secondary outcome measures include, the Modified Ashworth Scale (MAS), a 6-point scale that ranges from 0 to 4 where lower scores represent normal muscle tone and higher scores greater severity of spasticity [19]. ARAT, a 19-item observational measure used by physical therapists and other health care professionals assesses upper limb motor performance (coordination, dexterity and functioning). It is comprised of four subscales (grasp, grip, pinch and gross movement) and arranged in order of decreasing difficulty, with the most difficult task examined first, followed by the least difficult task [20]. The MCID for the ARAT is an improvement in the raw score of at least 4 points [21]. The Medical Research Council (MRC) strength scale will be recorded in the affected limb elbow flexor and extensors, from a score of 0 (no visible contraction) to a score of 5 (normal). The Leeds Adult Spasticity Impact Scale (LASIS) is used to assess the impact of spasticity on daily life. It is a measure of passive arm function that is administered by semi-structured interview to the patient or carer. It consists of 12 items of low difficulty that evaluate performance of daily functional tasks in the individual’s normal environment [22]. Quality of life assessments include the EQ-5D-5 L, which asks participants to rate their health in five dimensions: mobility, self-care, usual activities, pain/discomfort and anxiety / depression [23], and the Spasticity-Related Quality of Life 6-Dimensions (SQoL-6D), designed to assess quality of life in relation to upper limb spasticity across six domains (pain/discomfort; involuntary movements or spasms; restricted range of movement; caring for the affected limb; using the affected limb; mobility/balance) [24]. Each dimension is assessed using a five-level scale ranging from 0 to 4, with higher scores meaning worse condition. The Total SQoL-6D score is computed as a linear transformation of the mean of the six dimensions scores to have the Total score ranging from 0 to 100, with the direction of scoring inverted, so that a higher score indicates a better quality of life, in line with other instruments.

Healthcare utilisation is being assessed at baseline, EOT and at each follow-up visit at 6, 12, 24 weeks after end of treatment using a structured questionnaire developed for this study and supported by data abstracted from electronic health records (clinic visits, GP and hospital attendances, medications use).

### Safety reporting

While prior studies have not demonstrated any safety concerns associated with TENS of SHAPES in the management of spasticity [15, 16] safety outcomes will also be reported. The following definitions will be applied in the reporting of adverse events:

#### Adverse event (AE)

any untoward medical occurrence in a patient or clinical study subject. All such events, whether expected or not, will be recorded.

#### Serious adverse event (SAE)

any untoward and unexpected medical occurrence or effect that: (1) results in death, (2) is life threatening (event in which the subject was at risk of death); (3) requires hospitalisation, or prolongation of existing inpatients’ hospitalisation; (4) results in persistent or significant disability or incapacity; (5) causes a congenital anomaly or birth defect. All SAEs will be collected and recorded whether they are ‘related’, that is, resulted from the administration of any of the research procedures, or ‘unexpected’, that is, an event that is not an expected occurrence. In the event of any SAE unblinding will occur to establish potential causality.

### Pre-defined criteria for participant withdrawal

In the event that participants develop any of the following, the intervention will be stopped. Participants will continue to be assessed for outcome measures as per intention to treat analysis.

1. Patient requiring implantation of new electrical / electronic devices.
2. Patient experiencing a new stroke during intervention period.
3. Allergy to the electrodes or gel.
4. Incidental injury or dermatological condition over the extensor aspect of upper arm.
5. Significant medical illness interfering with delivery of intervention.
6. Pregnancy.

### Statistical analysis and sample size

Study analysis will be conducted on an intention to treat basis. Baseline characteristics will be presented by treatment group. Summary statistics for each of the endpoints will be reported at each available time point (baseline, EOT, and 6, 12 and 24 weeks after end of treatment). Results will be reported with confidence interval where relevant, for example, in reporting the average difference between treatments in the secondary endpoints. We previously carried out a community-based study using the NRS-S as an outcome measure [16]. Analysis of data from the study was used for the sample size calculation. A change in average NRS-S of 18% from baseline to follow-up time point (MCID) is considered a success. The proportion of successes will be compared between groups (pairwise).

A parallel gatekeeper design has been developed such that the first two significance tests share the total *p*-value equally (each tested at 0.025 level). This will be split equally between the first primary objective (SHAPES vs. usual care, and TENS vs. usual care at EOT (6 weeks) using 2.5% significance each). If one or both of these tests are significant then the *p*-value will be reutilised for testing the second primary objective (SHAPES vs. TENS at EOT and 6 weeks after EOT). This will utilise Chi-squared test (or an exact test if required). Between group efficacy at follow up timepoints (3 and 6 month) will also utilise a continuity corrected Chi-squared test.

We calculated the sample size using data from our previous study [15]. The sample size calculation for a two group t-test for equal means showed that to conduct a three arm randomized control trial of SHAPES, TENS and ‘usual care’ control, we would need 66 subjects per group (total 198 participants) to have 90% power to demonstrate a MCID reduction of 18% in NRS at the 1% significance level to allow for multiple testing. In our feasibility study 67% of participants showed good adherence with the intervention and 73% with recording of NRS. We will recruit a total of 297 patients with PSES to the study who are being randomised to one of the 3 arms. We aim to achieve a minimum of 66 participants to adhere to and complete the protocol in each group with good adherence to the intervention schedule and a minimum of 4 of 7 daily NRS-S recording. This will be known as the Per Protocol Set (PPS). Sensitivity analyses will be conducted to investigate the impact of missing or incomplete data, non-compliance, and spasticity severity. The sample size calculation was done in R using simulations.

Analysis of other secondary objectives (ARAT, MAS, MRC, LASIS, EQ-5D, SQoL-6D) will use repeated measures analysis with treatment group and timepoint as factors, baseline as a covariate and subject as a random effect to investigate the within-participant change in each outcome over time and to see if there is a treatment effect. As some of these outcomes are ordinal, it may not be possible to satisfy the conditions of a repeated measures ANOVA thus Friedman test may be used instead with pairwise Wilcoxon Signed Rank tests for any post-hoc analysis requirements. Since these analyses are exploratory, no adjustment for multiplicity will be made – nor will significant conclusions be drawn.

### Patient, public involvement and engagement (PPIE)

Service user involvement has been at the heart of developing the SHAPES intervention, from its origins in 2014 through to influencing the study duration and primary outcome measure and informing the usability for the final device being evaluated in this RCT. One of the contributing authors has lived experience as a stroke survivor and acts as PPIE advisor. Details of the co-design process and how this was undertaken during the COVID pandemic conditions have been described previously [14].

## Discussion

This study represents the largest trial conducted to date that investigates electrical stimulation therapies for spasticity. In particular the design allows generation of evidence for two differing electrical stimulation techniques, SHAPES and TENS. While TENS is already in widespread use for he management of spasticity after stroke, evidence for its efficacy is sparse, involving studies of small numbers of participants (*n* = 10 to 30) from varied populations including traumatic brain injury, multiple sclerosis and spinal cord injury in addition to stroke [25]. Establishing definitive evidence from a powered study such as SHAPES will help inform guidelines regarding its use. While it is often difficult to blind participants between electrical stimulation therapy and control, the electrical stimulator sleeve used in this study is very effective at blinding participants and researchers completing outcome measure assessments, to treatment allocation between the electrical stimulation techniques. The parallel health economic evaluation will also be critical in understanding how each type of therapy, if found to be effective for elbow spasticity, may be implemented in a UK healthcare setting. Because the SHAPES intervention is a relatively low cost, self-delivered intervention with the capacity for repeated long-term use, it has the potential to make a significant impact on spasticity management if found to be effective.

## Electronic supplementary material

Below is the link to the electronic supplementary material.

Supplementary Material 1

Supplementary Material 2

Supplementary Material 3

Supplementary Material 4

## Data Availability

The datasets collected and analysed during the current study will be available from the corresponding author on reasonable request.

## Abbreviations

TENS: Transcutaneous electrical stimulation
SHAPES: Sheffield Adaptive Patterned Electrical Stimulation
PSES: Post stroke elbow spasticity
NRS-S: Numerical Rating Scale
RCT: Randomised controlled trial
MAS: Modified Ashworth Score
UK: United Kingdom
ARAT: Action Research Arm Test
LASIS: Leeds Arm Spasticity Impact Scale
EQ-5D: European Quality of Life Scale 5D-5 L
SQoL-6D: Spasticity Specific Quality of Life Scale
MRC: Medical Research Council
MT: Motor threshold
NHS: National Health Service
AE: Adverse event
SAE: Serious adverse event
MCID: Minimally important clinical difference
EOT: End of treatment
PPIE: Patient, public involvement and engagement
