# Supplementary material for: Sheffield Adaptive Patterned Electrical Stimulation (SHAPES) Therapy for Post Stroke Arm spasticity: study protocol for a 3-arm, a partially blinded, randomised controlled trial
Source: BMC Neurol. 2024 Nov 9;24:437. doi: 10.1186/s12883-024-03635-x (PMC11549865; doi:10.1186/s12883-024-03635-x)
Supplement: Supplementary file 2 — Supplementary Material 2 [file 12883_2024_3635_MOESM2_ESM.pdf]

CENTRAL COMMISSIONING FACILITY
Grange House
15 Church Street
Twickenham
TW1 3NL

Tel: 020 8843 8000
Fax: 020 8843 8001
Email: ccf@nihr.ac.uk
www.nihr.ac.uk/ccf

Professor Wendy Tindale
Sheffield Teaching Hospitals
Floor I, Royal Hallamshire Hospital
Glossop Road
Sheffield
S10 2JF

19 February 2020

Dear Professor Tindale,

Invention for Innovation (i4i) Programme: NIHR201642 - A new therapy for post-stroke arm spasticity: Sheffield Adaptive Patterned Electrical Stimulation (SHAPES) - a co-designed system improvement followed by a powered multi-arm randomised control trial.

Your application to Call 19 of the NIHR Invention for Innovation Product Development Awards has been considered by the Committee.

We are pleased to inform you that you have been invited to submit a Stage 2 (full) application. On the following pages we will provide feedback from the Committee meeting and summarise the requirements and deadlines for submitting your Stage 2 application. Please read this document carefully and ensure you follow all instructions below.

Feedback on Your Stage 1 Application

The following points were raised by members of the Committee. Please address these carefully in your Stage 2 application.

- The Committee acknowledged that the unmet need was clearly articulated in the application.
- It was recognised that the project team is very experienced and has the appropriate expertise to deliver the proposed plan.
- The advantage over competitors was well described. It would have been interesting for the competitor analysis to also include products still under development, rather than only commercially available alternatives.
- Having support from the local CCG is particularly important in view of future adoption of the device.

- The project plan was well structured, and the trial should be sufficiently large to give a definitive outcome on efficacy.
- The Committee asked for more clarity on how the double blinding will be achieved, considering that patients in the control arm might not experience any stimulation.
- It would be beneficial at Stage 2 to include a description of the QALY parameters that will be employed. It was argued that self reported arm spasticity scores may be subject to reporter bias.
- The Committee agreed that a more detailed commercialisation plan should be described in the Stage 2 application.
- The project represents good value for money given the level of innovation and the potential for considerable cost savings to the NHS.
- Please note, the NIHR does not fund pieces of equipment whose value exceeds £5,000. Pieces of equipment costing more than £5,000 to purchase will usually need to be leased. Where applicants are leasing equipment with a purchase price of more than £5,000 a comparison of leasing versus purchasing costs must be provided in Section 11 under Justification of Costs.
- The plain English summary was clear but could be further simplified and freed from jargon to provide a better explanation of your research to a lay person. You may contact your local Research Design Service for further help. Input from patients may also help in improving the plain English summary.

Your application has also been reviewed by a member of the committee from the PPI perspective, and the following comments were provided. Please address these as part of your Stage 2 application:

- Early engagement with feasibility study participants and the Sheffield Stroke and Aphasia Research Public and Patient Involvement Group seems to be wholly appropriate.
- Work packages 2 and 3 both include ample PPI references with respect to study design, patient literature and training materials.
- It was thought to be important to ensure that PPI entails a broad and representative group of patients and carers, beyond those who might typically participate in PPI groups.
- The project team could consider including a lay person as a co-applicant to manage PPI activities in order to comply with INVOLVE standard 6.1 Governance.

Submission of Your Stage 2 Application

Please submit the following documents by the required deadlines and in the format described below.

- **Stage 2 Application:** Please submit your completed application by **1:00pm on 08 April 2020** through our Research Management System (RMS), addressing the feedback provided above. Please refer to our Guidance for Stage 2 Applications for further information on how to complete the online application form. Please pay particular attention to the requirements of Section 9, the Detailed Research Plan, and ensure that you clearly describe your IP and commercialisation strategy in this section.

- **Declaration and Signatures:** In order for your application to be accepted, your organisation is required to complete the declaration and signature form on the Research Management System (RMS). This must be completed before you submit your Stage 2 application.

Guidelines on Project Costs

Please read the i4i Finance Guidance document carefully and follow the guidelines. We would like you to pay particular attention to NHS support and treatment costs and discuss these with your local NHS trust and/or Local Clinical Research Network (LCRN). Please be aware that i4i does not fund NHS support or treatment costs; NHS support costs will be funded by the LCRN and NHS treatment costs will be funded by the local NHS trust.

Once you have submitted your Stage 2 application, you may be contacted by our Finance team at any time until the committee meeting to discuss any aspect of your project costs. It is important that you provide accurate information in a timely manner to ensure a smooth process.

Peer Review and Rebuttal

Your application will be subject to external review. You will be given the opportunity to respond to the peer reviewers' comments from approximately 18 May 2020**,** with a deadline of 25 May 2020.

Presentation to the Committee

You will be required to give a 10 minute presentation to the committee on **16 June 2020 at 2:30pm**, followed by a 15 minute Q&A session. These timings will be strictly adhered to and additional time will not be allowed. A list of all current committee members can be found on our website.

Please find attached a few tips on how to prepare your presentation for the committee meeting. Please submit details of the presenters (title, name, organisation, role in the project) to the i4i Programme by email (i4i@nihr.ac.uk) by **02 June 2020, 1:00pm.** Please note that a maximum of three members of the project team are allowed to attend the committee meeting. You are required to submit a copy of your full presentation, to the i4i Programme by email (i4i@nihr.ac.uk) by **09 June 2020, 1:00pm.** We are unable to accept any changes to your presentation after submission.

Please also note that your presentation will need to work through Microsoft PowerPoint 2010 and will be played via a Windows computer. If you wish to embed videos within your presentation, please ensure you let us know which slides these videos have been embedded into so that we can check they work on our system.

The committee meeting will be held at the **Central Commissioning Facility, Grange House, 15 Church Street, Twickenham TW1 3NL**. Please arrive at least 10 minutes before your scheduled presentation time and wait in the reception area. You will be greeted by one of the i4i Secretariat members who will accompany you to the meeting room.

Summary of Deadlines

Submission of Stage 2 application	08 April 2020, 1:00 pm
Response to peer reviews	25 May 2020, 1:00 pm
Submission of names of presenters	02 June 2020, 1:00 pm

Submission of presentation	09 June 2020, 1:00 pm
Committee meeting presentation	16 June 2020, 2:30 pm

Correspondence and Communication

Please note that during this process you will receive email correspondence through the RMS. Therefore please ensure that you check your email regularly and that the address manager@ccfrms.org.uk is not blocked by any firewall. If you have any questions, please do not hesitate to contact us.

Yours sincerely,

Dr Caterina Lombardo
Senior Programme Manager
E: i4i@nihr.ac.uk
T: 020 8843 8015
