# Supplementary material for: Sheffield Adaptive Patterned Electrical Stimulation (SHAPES) Therapy for Post Stroke Arm spasticity: study protocol for a 3-arm, a partially blinded, randomised controlled trial
Source: BMC Neurol. 2024 Nov 9;24:437. doi: 10.1186/s12883-024-03635-x (PMC11549865; doi:10.1186/s12883-024-03635-x)
Supplement: Supplementary file 3 — Supplementary Material 3 [file 12883_2024_3635_MOESM3_ESM.pdf]

NIHR | National Institute for Health Research

CENTRAL COMMISSIONING FACILITY
Grange House
15 Church Street
Twickenham
TW1 3NL

Tel: 020 8843 8000
Fax: 020 8843 8001
Email: ccf@nihr.ac.uk
www.nihr.ac.uk/ccf

Professor Wendy Tindale
Sheffield Teaching Hospitals
Floor I, Royal Hallamshire Hospital
Glossop Road
Sheffield
S10 2JF

06 July 2020

Dear Professor Tindale,

NIHR201642: A new therapy for post-stroke arm spasticity: Sheffield Adaptive Patterned Electrical Stimulation (SHAPES) - a co-designed system improvement followed by a powered multi-arm randomised control trial

I am pleased to inform you that the i4i Product Development Awards Funding Committee has recommended your application, submitted for consideration in Call 19, for funding. The Department of Health and Social Care, in their capacity as the National Institute for Health Research (NIHR), has confirmed their intention to award funding upon acceptance of the terms and conditions set out in the Standard Research Contract and pending agreement to the suggested amendments recommended by the i4i Product Development Awards Committee, as detailed in the Annex.

The Standard Research Contract, between Contractors and the Secretary of State for Health and Social Care for all initiatives can be found on the NIHR website at:

https://www.nihr.ac.uk/funding-and-support/funding-for-research-studies/manage-my-study/contracts-and-intellectual-property.htm.

Next Steps

The NIHR is committed to the rapid initiation of research following the decision to fund to benefit patients as soon as possible. Therefore, we expect funded researchers to be working towards gaining the necessary contractual agreements and governance approvals required to start the project within 6 months or by a date mutually agreed by both parties on acceptance of the award.

The NIHR acknowledges the risk to organisations around committing resource to research before a contract is in place; however, it is rare to not reach contractual terms unless the circumstance of the research team changes. The NIHR, therefore, encourages organisations

to commit staff to setting up projects at as early an opportunity as possible in order to expedite the formal commencement of research.

It is acknowledged that there can be unforeseen delays in starting up a research project, but in order to help reduce these it is your responsibility to work closely with your organisation's R&D department or equivalent as well as other colleagues/departments involved in the administration and management of the research, and to start these discussions at the earliest opportunity.

To ensure that the project starts within the agreed timeframe with all the required agreements and approvals in place, appropriate staff (such as project and/or study managers) need to be in post as early as possible after receiving this letter of intent. These staff costs will ultimately be covered through the research funding award, but you are encouraged to meet them from Research Capability Funding (RCF) or similar resources, where available, prior to the research contract being agreed.

To support the often-iterative process towards agreement of the contract, we have set out the guiding timeframes for the submission of responses or information for each step towards the agreement of the Standard Research Contract as well as the anticipated start date.

- Confirmation of acceptance of funding – no later than 1 week from date of letter
- Responses to Committee feedback and queries – within 3 weeks of receipt of the queries
- Responses to Finance and IP queries – within 3 weeks of receipt of the queries
- Contract signature – within 4 months from date of this letter
- Contracted commencement start – within 6 months from date of this letter or a date mutually agreed on acceptance of award
- Submission of draft collaboration agreements and/or subcontracts (where applicable) – within 1 month of project start

On receipt of information as set out above, the NIHR through the Central Commissioning Facility is committed to responding to your submission of information within two weeks or we will update you on progress.

Please take the time to carefully read the enclosures to this letter which details the feedback on your application, your contact within the Central Commissioning Facility who will be working with you on the contract, the processes to be undertaken during the next steps, as well as additional information relating to your award.

Yours sincerely,

Dr David King
Director, Central Commissioning Facility

Enclosures:
- Contact details of CCF staff as next contact
- Feedback from Committee
- Queries from i4i Secretariat
- NIHR Clinical Research Network (CRN) support
- HRA approval

cc: Dr Dipak Patel (dipak.patel12@nhs.net)

ANNEX

Contact details of CCF staff as next contact

Dr Caterina Lombardo, Senior Programme Manager

E: caterina.lombardo@nihr.ac.uk

T: +442088438139

Feedback from Committee

- The Committee agreed that this is overall a good proposal. However, there was concern around the timelines associated with the clinical trial, in particular regarding the last follow-up being at 3 months. Applicants are invited to reconsider trial timelines.
- The Committee was pleased that the team was awarded an EPSRC grant to investigate the mechanism of action of this innovation, as this will complement well the knowledge that will be acquired during the NIHR i4i project.
- The approach to commercialisation and adoption presented in the application and discussed during the interview was considered sensible. The Committee recognised that the team has experience in developing products focusing on NHS and patients' needs, and has the right connections to demonstrate demand for this technology.
- The PPI was considered excellent.

Queries from i4i Secretariat

<u>Administrative</u>

1. Please confirm the proposed start date of 01 October 2020. Please note that NIHR projects must start on the 1st of each month.
2. Please confirm that the personnel involved in the project are still those stated within the application.
3. Please provide a draft collaboration agreement between the following project parties; Sheffield Teaching Hospitals NHS Foundation Trust, Royal Hallamshire Hospital, Coventry University, University of Sheffield and Barnsley Hospital NHS Foundation Trust. N.B. the delivery of a fully executed agreement may be part of a milestone which must be completed within 3 months of project start. The NIHR will need to review the agreement/s before signature.
4. Please use the below timetable to suggest go/no-go points within the project.

WP #	Work Package Title	Milestones (Y/N + detail)	Go/ No-Go

	Please add extra rows as necessary		

Finance

1. Please propose a suitable payment schedule. Please note that payments are made quarterly in arrears.
2. An amount of £71,046 was requested for a research nurse. Please define which activities have been classed within the research cost package.
3. Regarding the inspection microscope - please explain the purpose of the device. Is this for screening patients?
4. Note that the NIHR operates a strict limit on PC/laptops of £650 per unit. Please confirm that the computer to be acquired for computer aided design work, will be under the cost limit.

IP and Commercial

1. Please complete the table below in relation to any background IP associated with the project. If none, please mark N/A.

**Description of Background IP**	**Owner of relevant Background IP**	**Nature of restriction**	**Risk to Research and outcomes**

2. Please complete the table below in relation to any foreground IP that may be generated by the project.

**Description of Foreground IP**	**Owner of relevant Foreground IP**	**Is it severable or non-severable?**

NIHR Clinical Research Network (CRN) Support

If your study involves the NHS or NHS patients, we expect you to apply, where appropriate, for NIHR CRN support and subsequent inclusion in the NIHR CRN Portfolio of studies to fully benefit from the support that the CRN offers through their Study Support Service. To find out more, please visit http://www.supportmystudy.nihr.ac.uk.

HRA Approval
If your study involves NHS sites in England you will need to apply for Health Research Authority Approval and follow the appropriate process for setting up sites. Please visit: http://www.hra.nhs.uk.

Publicity
Please be aware that no publicity concerning this award should be undertaken prior to exchange of contract agreement, and without full involvement of CCF's Communications team. Please liaise with your Senior Programme Manager in the first instance on any issues relating to publicity.
