# Supplementary material for: Sheffield Adaptive Patterned Electrical Stimulation (SHAPES) Therapy for Post Stroke Arm spasticity: study protocol for a 3-arm, a partially blinded, randomised controlled trial
Source: BMC Neurol. 2024 Nov 9;24:437. doi: 10.1186/s12883-024-03635-x (PMC11549865; doi:10.1186/s12883-024-03635-x)
Supplement: Supplementary file 4 — Supplementary Material 4 [file 12883_2024_3635_MOESM4_ESM.pdf]

NIHR | National Institute for Health Research

NIHR Invention for Innovation (i4i)

Applicant Response to Peer Reviewer Comments

Reference number	NIHR201642
Lead Applicant	Professor Wendy Tindale

Please enter your response below (Maximum of 3300 words).

We thank the reviewers for their thoroughness and constructive comments. We are pleased that the majority are supportive of the project. Any points that we have not addressed we will do so at interview. Specific points are addressed below:

1. **RELEVANCE OF THE PROPOSED WORK**

Reviewer-1

Comment**:** *Spasticity occurs in a minority of stroke survivors (as the authors acknowledge ~17%).*
Response**:** We did not cite ~17%, we gave the published range of spasticity occurrence data: i.e.17%-43% of stroke survivors annually.

Comment**:** *Spasticity rarely develops in the short term after stroke, it is usually becomes troublesome (to the point that it needs to be treated) several weeks or months after stroke, so there is little value in recruiting two weeks post stroke*
Response***:*** We intend to recruit participants from 2 to 16 weeks post-stroke. Regrettably there was an error on page 7 (‘at’ instead of ‘from’ that could have been misleading). However, we had been clear on p13 that the recruitment would be from 2 weeks and spanning the rehabilitation pathway.
Comment: *Stroke survivors who develop troublesome spasticity almost invariably have severe weakness. This is the primary cause of disability, not the spasticity.*
Response: We are well aware of this association and arm weakness is an inclusion criterion along with a spasticity MAS score of at least 1. We believe that addressing the spasticity will result in an associated improvement in function as the two are inter-related.
Comment: *The applicants raise several issues with the use of botox but give no explanation about how SHAPES would address them.*
Response: The reviewer states that Botox is cost-effective, according to RCP Guidelines. This not yet evidenced. The guidelines state that it has the potential to be cost-efficient if given by non-medical prescribers. Relating to its use in upper limb PSS, the NIHR BoTULS RCT stated ‘*no evidence to suggest that botulinum toxin type A plus therapy is a cost-effective alternative to therapy alone in this patient group*’. As reviewer-1, notes, drug costs for each injection are ~£400, with and must be repeated every ~12 weeks to remain effective and its therapeutic effects are not uniform over time. SHAPES is a reusable device with an upfront one-off cost. It can be self-managed at home without need for out-patient hospital visits, unlike Botox.
Comment: applying to one set of muscles (elbow) will have limited application.
Response: Most TENS is used in single channel form and applied to one set of muscles. The elbow is affected in 79% of those with PSS (79%) -Wissel, 2010 in our submission. We expect SHAPES to have future multi-joint application, but a trial against
Comment: If using varying frequencies (typically 10-100Hz) habituation doesn't occur.
Response: We can find no published evidence to support this comment. Ones addressing frequency modulation for pain [1,4] show some reduced habitation over short 20min periods.

Reviewers-2to5:

We thank reviewers 2-5 for their positive comments:

- *need for home based rehabilitation post stroke is urgent and well known.*
- *aligns well with research NHS England priorities*
- *the result will add to knowledge of the treatment of spasticity after stroke*

QUALITY OF THE PROPOSED WORK

Reviewer-1

Comment:

- Disagrees with the primary objective – stating that a reduction in arm spasticity won’t improve arm function.

Response**:**

- Recruitment is for people with arm weakness and arm spasticity and outcome measures assess both.

Reviewer-1:
Comment: Couldn't see where *'real-time adherence and movement monitoring from the device be of clinical benefit or design of new electrode array offering concurrent elbow and wrist stimulation*' were mentioned.
Response:
- Adherence monitoring is provided by the device indicating retrospectively when it has been active and in use. Its benefits will be reviewed with the PPI group.
- We considered a two joint configuration for the trial and decided to demonstrate effectiveness in one joint before progressing to trials of two or more. The new electrode array will permit future concurrent stimulation of **multiple arm joints** (e.g. elbow and wrist).

Reviewer-2

Comment: Queried that the cited stroke numbers were too low.
Response: We stated 8,670 to 21,930 and should have made clear that this is those with post-stroke elbow spasticity.)

Mechanism
We hypothesise that the effects of TENS on spasticity can be enhanced by the SHAPES stimulus that has two distinct features (Slovak, 2016 in proposal).
- **Stimulation of a larger area of skin to hence stimulate more sensory fibres**. This could be achieved using larger TENS electrodes. However, the stimulus current density would not be guaranteed to be distributed evenly over the electrode and, in particular, would be expected to be greater at the edges of the electrode (Reilly, 1992). The SHAPES 64-channel electrode array allows **delivery of stimuli evenly over a larger area** compared to TENS.
- **Stimulation of a larger area of skin to hence stimulate more sensory fibres**. This could be achieved using larger TENS electrodes. However, the stimulus current density would not be guaranteed [is it 'would not be guaranteed' or is it 'would be unlikely to'?] to be distributed evenly over the electrode and, in particular, would be expected to be greater at the edges of the electrode (Reilly, 1992). The SHAPES 64-channel electrode array allows **delivery of stimuli evenly over a larger area** compared to TENS.
- Reciprocal inhibition of antagonist muscle groups plays an important role in voluntary movement in healthy subjects. Spasticity is believed to result from impairment to this mechanism – particularly of 1a sensory fibres. **Patterned sensory stimulation is more effective in inducing plasticity in reciprocal inhibition** (Crone, 1994a,b).

RCT Design:
The clinical trial design as intended (and detailed within the SoECAT) is for the three arms to be 1) TENS+ usual care, 2) SHAPES+ usual care and 3) usual care only. We trust that this clarification will address Reviewer 2's understandable concern regarding ethics. We regret that we had not made this clearer in the main body of the proposal
Reviewer-1:
Comment: *The application is a classic example of technology that has been developed for the sake of the technology and then has been 'retrofitted' to try to find a clinical application.*

Response: We do not know on what evidence this assertion was based. The evolution of this project arose from clinical need, and at the request of a clinicians and patients for new ways of treating spasticity. We can assure the i4i team that the Devices for Dignity MIC as an NIHR-funded organisation works on the basis of responding to unmet clinical need. We do not develop technology for technology's sake.
Outcome measures:
Comment:
Reviewer 1 advised that we should use the Arm-A questionnaire as our primary outcome measure instead of the NRS for spasticity.
Response: We are open to alternatives. We note that the Arm-A questionnaire consists of a 21-item questionnaire (self-care and functional tasks) intended to be competed weekly compared to the single NRS-Spasticity visual analogue score intended for daily completion. We do not think that it would be appropriate as the primary outcome measure but will seek the opinion of our PPI advisors regarding substituting it for the similar
Leeds Adult Spasticity Impact Scale (LASIS). Unlike the NRS, we have not trialled the Arm-A previously.
Comment: The applicants also propose to use *an un-validated measure of 'self-determined patient-specific functional goals' on the basis that other researchers have used it.*
Response:
- We have not stated this in the application and believe that reviewer 1 has conflated two separate measures, the SQoL-6D and p*atient-specific functional goals.* The SQoL-6D from Kings College London is referenced in RCP Guidelines and is being validating in a large European study. We plan to review it in advance of the

project as a potential additional secondary outcome measure to that of the EQ-5D for quality of life (QoL) (page 10 of application) because the S-QoL-6D is a spasticity-specific QoL tool.
- Regarding *patient-specific functional goals*, we had included this outcome in direct response to patient feedback. We were not referring to an '*un-validated questionnaire*'. We had not specified the process for goal measurement and will consider the Goal Attainment Scaling as suggested by Reviewer 1, although we note from the RCP 'Spasticity in Adults' guidance that it has had problems in clinical use. The modified GAS-light may be preferable and we will investigate the appropriateness of its use

Reviewer-2

Comment : Q*uality assurance of the RCT unclear*
- We will have a Trial Steering Committee with an independent Chair (invited from University of Sheffield Clinical Trials Research Unit (CTRU); a Data Monitoring and Ethics Committee with independent Chair and Statistician; and advisory input from the CTRU.

Response:
- We will employ online **OpenClinica** for our data management, including standard forms for data entry checking, and randomisation allocation.
- We will have a statistical analysis plan in place before soft-lock of the trial database.

Various comments related to the **TRIAL PROTOCOL**; we include a **truncated version** below:

Protocol:

We will use a 3-arm RCT design. The **stimulation arms of the trial are double blind** as the participant and assessor will be blinded to the intervention; the control arm is un-blinded. Investigators pre-selecting TENS or SHAPES protocols on the devices in accordance with the randomisation will be independent of the investigators visiting participants.

Sample size: The sample size calculation for a two group t-test for equal means showed that to conduct a three-arm randomized control trial of 'TENS + usual care' (group-1) 'SHAPES + usual care' (group-2) and 'usual care only' (group-3) control, we would need 68 subjects per group (total 204) to have 90% power to demonstrate a minimal clinically important difference (MCID) of 18% in NRS at the 1% significance level to allow for multiple testing. The selection of 18% MCID is based on published work by Farrar [13].

Recruitment: Sheffield Teaching Hospitals is the regional centre for stroke. Our Sentinel Stroke National Audit Programme audit data for 2018-2019 showed that 935 patients were admitted over 12 months of which 807 survived and were discharged. 624 had upper limb weakness and spasticity. The rate of consent for our previous trials was 70% with protocol adherence rates of 63%. Thus, we need to approach 468 stroke survivors, to achieve 327 agreeing to participate and the 204 completing with good adherence to treatment required for the powered trial.

Participants: 327 men and women will be randomised to receive usual care, SHAPES with usual care or TENS with usual care. Participants will be identified from acute stroke wards, stroke rehabilitation wards, community stroke rehabilitation centres, physiotherapy services, the stroke spasticity clinic and the Functional Electrical Stimulation clinic. The treating team will identify the potential participants and give them the study information leaflet. A member of the study team will then contact potential participants 7-14 days post identification. Those who agree to participate will be invited to an appointment (Visit 1) with a member of the study team.

Visit-1 (week 1): The participant will be given an opportunity to discuss the study and any concerns with the lead study clinician. A member of the research team will then seek informed consent, if obtained the participant will be screened using the following criteria:

Inclusion Criteria: 1) Age 18 and above; (2) 2-16 weeks after stroke (3) Weakness of elbow extension of MRC grade 4 below (4) Spasticity of elbow, of grade-1 or more on the modified Ashworth scale of elbow flexion.

Exclusion criteria: (1) Dermatological, rheumatologic or orthopaedic illnesses of the affected arm interfering with elbow movement (2) Pre-existing severe systemic disorders like cardiovascular disease, active cancer or renal disease, end stage pulmonary or cardiovascular disease, psychiatric illness including severe alcohol or drug abuse and depression, (3) Inability to perform the baseline assessments, (4) Severe tactile hypersensitivity, (5) Participation in other, spasticity related studies (6) Within 12 weeks of receiving Botulinum toxin injections,(7) Uncontrolled epilepsy (8) Pacemaker or any other implanted devices (9) Pregnancy.

The participant or carer will be trained to record the severity of spasticity in a daily diary on a scale ranging from 0 to 10; 0- no spasticity, 10 worst spasticity you can imagine. The participant will be randomly allocated to group-1, group-2 or group-3 using an online randomisation system.

Interventions:

Participants in Groups-1 and 2 will receive the intervention for 60 minutes daily for six weeks, in addition to usual care. Researcher-1 who is blind to patient allocation groups will perform the baseline and outcome assessments. Researcher-2 will determine and record the maximum tolerable level of stimulation. Researcher-2 will demonstrate to the participant and/or the carer how to apply the stimulation and will instruct the participant.

In both Groups1 and 2, the SHAPES 64-channel (8x8) array will be placed over the extensor aspect of the affected upper arm and used to deliver either the TENS or SHAPES stimulation. At the start of first session, stimulation will be increased over the arrays until movement is first observed. The stimulation level will then be set at 90% of this value, or the maximum tolerable, if discomfort is reported and the value recorded. TENS or SHAPES stimulation will be applied at this perceived level for 60 minutes daily for 6 weeks. A researcher will aid in delivering the intervention

for participants in hospital or community rehabilitation unit at the time of recruitment. The participant/carer will be trained to deliver the intervention before discharge.
TENS group (Group-1): The SHAPES device will be pre-programmed to deliver non-spatially varying TENS stimulation to the central part of the array spanning a standard 5x5cm electrode.
SHAPES group (Group-2): Will receive SHAPES stimulation as 1 row of 8 channels at a time, moving rows 1 to 8, proximal to distal to simulate a stroking sensation.
Control group (Group-3)*:* Continue with usual care.

Assessments: Assessments will be performed prior to the start of, at the end of treatment phase and 12 weeks after completing the intervention.
Primary outcome is:

- elbow spasticity after six weeks of intervention measured by the Numerical Rating Scale (NRS),

Secondary outcome measures are:

- Action Research Arm Test,
- Measures of muscle tone (Modified Ashworth Scale),
- Strength (Medical Research Council grading),
- Impact of spasticity (Leeds Arm Spasticity Scale-reference),
- Quality-of-life (EQ-5D-5L),
- Qualitative interviews and questionnaire designed to capture patient's perception towards efficacy and acceptability of treatment.

4. Strength of the research team
3. STRENGTH OF THE RESEARCH TEAM
Reviewer-1: *The team covers most bases and seems to have a lot of relevant experience and connections.*
Comment: However, the reviewer held a strong view that: *expertise in clinical trials of rehabilitation / complex interventions was lacking.*
Response:
We were surprised at this comment as the team includes 2 highly experienced research-active Consultant neurologists (Dr Nair, co-applicant clinical lead and Dr Ali, a named collaborator and deputy clinical lead) who have led clinical trials (including 2 that precede this application) and two research physiotherapists. The project and proposal has been discussed with the NIHR Yorkshire and Humber Research Design Service who are supportive of our approach. We also have involvement of the University of Sheffield School of Health and Related Research (which hosts a NIHR CTRU), through Professor Simon Dixon at the University of Sheffield, the NIHR Devices for Dignity (D4D) MIC and the Sheffield Institute for Translational Neuroscience through Dr Ali, and D4D's long-term neurological conditions theme lead, Professor Chris McDermott.. The D4D team (including the CI and co-applicants) have been instrumental in the success of a previous NIHR i4i project 'Head Up' for neurological neck muscle weakness with a completion of a 150 participant multi-centre trial that led to a commercial product. D4D co-applicants/named collaborators also continue to support the technology strand of the NIHR Programme Grant funded multi-centre DAFNEplus multi-centre trial of a complex intervention for type 1 diabetes.
Reviewer-2:
Team has a wealth of experience and relevant expertise. Comments that '*might have been useful to include a physio co-applicant*'.

- We named the maximum number of co-applicants allowable. We do have 2 neurology specialist research physiotherapists costed in and we will have an independent experienced physiotherapist on our advisory committee.

Reviewer-3: *The research team is strong and broad based. They have an excellent track record in medical device development and investigation. I have no concerns in this regard.*
Reviewer-4: *Confident that the research team have all the requirements*. Does refer to a query re: RCT which we address above.
Reviewer 5: We thank he/she for the kind comment **'***I cannot think of any review I have completed has shown such a strong team*'.

4. IMPACT OF THE PROPOSED WORK
Reviewer-1 is unclear of the project impact
Reviewer-2 In contrast, was clear on the impact but wanted information on the physiological mechanism, and a more detailed project follow-up.

Response: Accumulated experimental evidence has supported supraspinal origins of spasticity, likely from an imbalance between descending inhibitory and facilitatory regulation of spinal stretch reflexes secondary to cortical disinhibition after stroke. (Li and Francisco, 2015; 6). We believe that we have addressed the SHAPES anticipated physiological mechanism earlier in the rebuttal, and in prior published work, (Slovak, 2016).

Reviewer-3: The project is well described and feasible, but obviously the outcome can't be guaranteed.
Reviewer-4: The applicants have clearly addressed all the areas for this section

Reviewer-5: Again we are pleased that the lay reviewer, a stroke survivor, considers the project to be a '*No brainer*' and states '*I am impressed*'.

5. VALUE FOR MONEY

Reviewer-1 felt unable to judge value for money (based on perceived clinical uncertainties).

Reviewer-2 commented that requested costs and resources were reasonable. Two queries were raised:

1. Whether the £2k device cost was final retail price.
    a. The £2k cost was extrapolated from the production costs of small scale clinical prototype systems. In volume production the costs would be closer to that of TENS devices. An alternative approach would be a free 3-month trial to confirm responder status to de-risk followed by quarterly leasing to remove purchase costs. This an approach used for other stimulator devices recently given positive guidance recommendation by NICE MTAC.
2. We agree that a TENS device can be purchased for ~£60 (e.g. MultiTrac, £62 used in our previous studies) and note the following:
    a. The MultiTrac TENS device is dual channel, thus each channel costs £31. The SHAPES device provides 64-independently programmable channels of stimulation, even at the low-volume prototype production price of £2,000 offers an equivalent £31 per channel cost and we expect this to reduce significantly.
    b. TENS devices are not yet regulated for routine upper limb spasticity therapy due to lack of robust evidence – they are primarily marketed as pain relief devices.

<u>Comment:</u> Related to engineering software costs, suggesting that University overheads could be used to cover them.

<u>Response:</u> The engineering is being done within an NHS department where only direct costs are allowable.

In contrast to Reviewer 1,

Reviewer 3 commented that resources requested were **clearly justified** and **Reviewers 4 and 5** comment that the project offers **excellent** and **good value for money** respectively.

6. INVOLVEMENT OF PATIENTS AND THE PUBLIC

We are delighted that the reviewers have unanimously praised the quality of our involvement of patients and the public with comments such as 'excellent PPI', 'PPI is fully invested',

7. ADDITIONAL COMMENTS

Reviewer-2: We note the comment '*I think this is an interesting idea that needs more basic research on the physiological mechanism and would recommend a change to the clinical trial design'*.

- In parallel we plan to undertake more basic research in this area through a funded EPSRC project due to start in March 2021
- We believe that we have addressed the RCT design earlier in the rebuttal. .

Reviewer-3: We note the reviewer's request (who accepts it not being in the scope of the proposal) for the addition of mechanistic background and offer the following:

- We believe that we have addressed this earlier in the rebuttal

Reviewer-4: We thank the reviewer for stating '*This is an important project in the field of clinical interventions for elbow spasticity after stroke*'

Reviewer-5: We are pleased that the lay reviewer added '*I can see a huge positive if this application succeeds'*

ADDITIONAL REFERENCES: NIHR201642

1. Avendaño-Coy J., Bravo-Esteban E, Ferri-Morales A., Martínez-de la Cruz, R., and Gómez-Soriano, J. Does Frequency Modulation of Transcutaneous Electrical Nerve Stimulation Affect Habituation and Mechanical Hypoalgesia? A Randomized, Double-Blind, Sham-Controlled Crossover Trial. *Phys Ther*. 2019, 99:924–932.
2. Cavalcanti Garcia, M.A. and Domingues Vargas, C. Is somatosensory electrical stimulation effective in relieving spasticity? A systematic review. *J Musculoskelet Neuronal Interact* 2019; 19(3):317-325
3. Serrano-Muñoz D, GoÂmez-Soriano J, Bravo-Esteban E, VaÂzquez-Fariñas M, Taylor J, Avendaño-Coy J. Intensity matters: Therapist-dependent dose of spinal transcutaneous electrical nerve stimulation. (2017). *PLoS ONE* 12(12): e0189734. https://doi.org/10.1371/journal.pone.0189734
4. Spielholz, N. I. and Nolan,M.F. Conventional TENS and the *Phenomena of Accommodation, Adaptation, Habituation, and Electrode Polarization. J Clin Electrophysio*. 1995, 7:16-19
5. Stein, C. Gassen Fritsch, C. Robinson, C., Sbruzzi, G., and Della Méa Plentz, R. Effects of Electrical Stimulation in Spastic Muscles After Stroke. Systematic Review and Meta-Analysis of Randomized Controlled Trials. *Stroke*, 2015, 46(8):2197-2205 https://doi.org/10.1161/STROKEAHA.115.009633/
6. Li, S. and Francisco, G.E. New insights into the pathophysiology of post-stroke spasticity. Front Hum *Neurosci.* 2015; 9:192. https://www.ncbi.nlm.nih.gov/pmc/articles/PMC4392691/
7. Christofi, G, Ashford, S, Birns J., Dalton, C. Duke, L. Madsen,C and Salem S. Improving the Management of Post Stroke Spasticity: Time for Action, J*. Rehab Med* 2018; 1: 1000004: https://www.medicaljournals.se/jrm/content/html/10.2340/20030711-1000004
8. Welmer, A-K, Holmqvist, L. Sommerfeld, D. Location and severity of spasticity in the first 1-2 weeks and at 3 and 18 months after stroke. *European journal of neurology*, 2010, 17:5:720-725. https://doi.org/**10.1111/j.1468-1331.2009.02915.x**
9. Reilly JP. Electrical stimulation and electropathology. Cambridge, UK: Cambridge University Press, 1992: 28.
10. Crone C, Nielsen J. Central control of disynaptic reciprocal inhibition in humans *Acta Physiol Scand* 1994;152:351–363.
11. Crone C, Nielsen J, Petersen N, Ballegaard M, Hultborn H. Disynaptic reciprocal inhibition of ankle extensors in spastic patients. *Brain* 1994;117(Pt 5):1161–1168.
12. Rattay, F. 1988. Modeling the excitation of fibers under surface electrodes. *IEEE Trans Biomed Eng*,35,199-202.
13. Farrar JT, Troxel AB, Stott C, Duncombe P, Jensen MP. Validity, reliability, and clinical importance of change in a 0-10 numeric rating scale measure of spasticity: a post hoc analysis of a randomized, double-blind, placebo-controlled trial. *Clinical therapeutics*. May 2008;30(5):974-985.
14. Reeves ML, Chotiyarnwong C, Nair KPS, Slovak M, Healey TJ., McCarthy AD, Patterson L, Lavender K, Strachan L, Ali, AL, Baster K. Caregiver Delivered Sensory Electrical Stimulation for Post Stroke Upper Limb Spasticity: A Single Blind Crossover Randomized Feasibility Study. *Health and Technology* https://doi.org/10.1007/s12553-020-00436-3
